# Verruca Vulgaris Occurring on a Tattoo: Case Report and Review of Tattoo-Associated Human Papillomavirus Infections

**DOI:** 10.7759/cureus.17575

**Published:** 2021-08-30

**Authors:** Philip R Cohen

**Affiliations:** 1 Dermatology, University of California, Davis Medical Center, Sacramento, USA

**Keywords:** epidermodysplasia, human, infection, papillomavirus, plana, tattoo, verruca, verruciformis, viral, vulgaris

## Abstract

Cutaneous infections can occur on tattoos. Tattoo-associated viral infections can be caused by human papillomavirus. A verruca vulgaris developed on the tattoo of a 44-year-old woman; the viral lesion appeared 21 years after she received the tattoo and had been increasing in size during the prior five years. Biopsy of the lesion not only confirmed the diagnosis but also removed most of the wart; the patient declined any additional treatment. In addition to verruca vulgaris (27 individuals), verruca plana (14 individuals) and human immunodeficiency virus-associated acquired epidermodysplasia verruciformis (two men) are human papillomavirus lesions that have been observed to occur on tattoos. The latency period from receiving the tattoo to the appearance of the wart has ranged from one month to 21 years; the median duration was 21 months for verruca vulgaris and 24 months for verruca plana. The warts most frequently appeared in the dark, usually black, inked areas of the tattoo; indeed, it has been postulated that the ink created a cutaneous immunocompromised district that enhanced the opportunity for the viral lesions to occur in the tattoo. The use of contaminated instruments or ink during tattoo inoculation is the most likely etiology for the development of a wart on a tattoo. However, other potential mechanisms for human papillomavirus to occur on a tattoo include transmission of the virus from the tattoo artist’s ungloved hand or saliva, a preexisting (albeit unrecognized) human papillomavirus lesion adjacent to or at the site of the tattoo, and postinoculation acquisition of the verruca at the site of the tattoo. Topical retinoid or imiquimod, used as a single agent, was not effective in the treatment of the warts. Some of the patients who were treated with cryotherapy using liquid nitrogen did not achieve any improvement of their viral lesions. However, other patients observed resolution of most or all their warts when cryotherapy with liquid nitrogen, either as monotherapy or followed by topical application of 5% imiquimod cream, was used; yet, following treatment, these individuals experienced mild distortion of their tattoo and/or hypopigmentation. Curettage and squaric acid dibutyl ester contact immunotherapy were both successful approaches to the management of tattoo-associated warts. In addition, warts were efficaciously managed with either photodynamic therapy or treatment with an ablative erbium:yttrium aluminum garnet (YAG) laser followed by topical application of 5% imiquimod cream.

## Introduction

Tattoos are a form of decorative body art. They are created by inoculating pigment dyes of various colors into the skin. Several adverse cutaneous events have been associated with tattoos [1,2].

Verruca vulgaris is a viral infection caused by human papillomavirus. More than 200 serotypes of human papillomavirus have been identified. Warts are contagious not only to the individual who has the wart by autoinoculation but also to other people to whom the infected person contacts [1,3].

A 44-year-old woman developed a nodule on her lower leg in an area which contained a tattoo. A biopsy of the lesion established the diagnosis of verruca vulgaris. The occurrence of human papillomavirus infection at the site of a tattoo is reviewed.

## Case presentation

A 44-year-old woman presented with a new lesion in her tattoo. Her medical history was significant for hypertension and hypothyroidism. She was currently taking the following medications: lisinopril and levothyroxine.

Her tattoo was received when she was 18 years old; this was 26 years ago. The asymptomatic lesion, which developed within her tattoo, appeared five years ago. It has subsequently increased in size.

**Figure 1:**
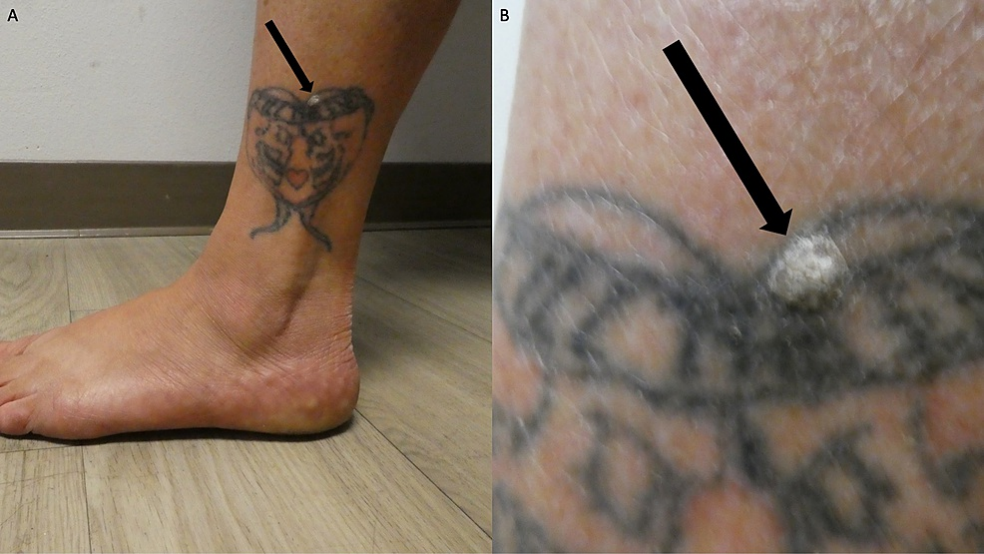
A single cutaneous lesion that contains both a verruca vulgaris and a tattoo Distant (A) and closer (B) views of the left ankle of a 44-year-old woman that shows a nodular skin lesion (black arrow) that has developed in the black tattoo.

Cutaneous examination showed a tattoo on the distal and lateral left leg proximal to her ankle. Within the superior portion of the tattoo, there was a painless, gray-white, 5 × 7 mm nodule. The lesion was located within the black pigment of her tattoo (Figure 1A, 1B).

**Figure 2:**
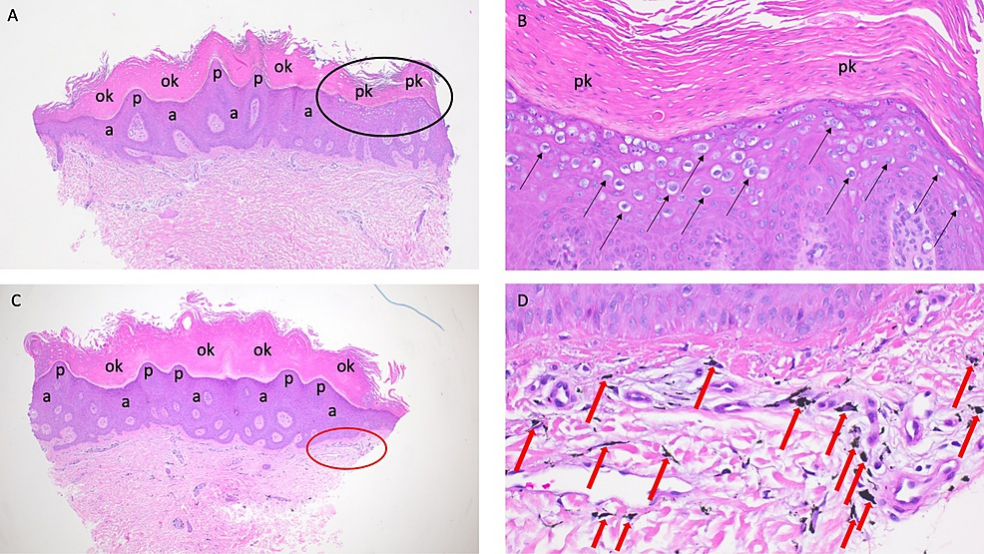
Microscopic features of a skin lesion that contains both a verruca vulgaris and a tattoo The 3-mm tissue biopsy specimen of the lesion was bisected. One-half of the specimen (A and B) shows low (A) and higher (B) magnification views demonstrating salient features of the wart. There is overlying hyperkeratosis of the stratum corneum consisting of both orthokeratosis (ok) and parakeratosis (pk) acanthosis (a) of the epidermis. There is thickening of the epidermis (a) and an irregular undulating configuration of the epidermis [papillomatosis (p)]. The area within the black oval of A is shown in B; in the upper layers of the epidermis, there are numerous vacuolated keratinocytes [koilocytes (black arrows)]. The other half of the specimen (C and D) shows low (C) and higher (D) magnification views demonstrating not only features of the wart (such as ok, a, and p) but also the tattoo. The area in the red oval of C is shown in D; some of the tattoo pigment (which appears as black amorphous material in the upper dermis) is demarcated by the red arrows (hematoxylin and eosin: (A) ×4; (B) ×20; (C) ×4; (D) ×40).

Microscopic examination showed compact thickening of the stratum corneum (hyperkeratosis) that consisted of both orthokeratosis (featuring cells without nuclei) and parakeratosis (characterized by cells with retained nuclei). There was thickening (acanthosis) and irregular undulation (papillomatosis) of the epidermis. Evidence of human papillomavirus infection was demonstrated by the vacuolated keratinocytes (koilocytes) that were abundantly present in the upper layers of the epidermis. In the upper dermis, amorphous exogenous black pigment (diagnostic of tattoo) was present (Figure 2A-2D).

Correlation of the clinical presentation and pathology findings established the diagnosis of verruca vulgaris at the same cutaneous location as her tattoo. The patient declined any additional treatment since the biopsy had removed most of the wart.

## Discussion

Infections may occur at the site of tattoo. The cutaneous infections can result from bacterial, fungal, mycobacterial, protozoan, spirochetal, and viral etiologies. Viral infections include those caused by human papillomavirus: verruca vulgaris, verruca plana, and epidermodysplasia verruciform [1-20]. 

Verruca vulgaris is a common manifestation of human papillomavirus infection. It typically occurs in immunocompetent individuals who are often unaware of the source of their contagious infection. However, when human papillomavirus infection presents in the genital region as condyloma, it is usually acquired as a sexually transmitted disease and the affected individual may concurrently have either other systemic viral diseases such as hepatitis B, hepatitis C, human immunodeficiency virus or spirochetal disease such as syphilis or both [2,3].

Verruca vulgaris on a tattoo, to the best of my knowledge and including the patient in this report, has been reported in 27 individuals (Tables 1, 2) [2-10]. The first patient was described in 1884 as a case of warts occurring on tattooed lines. An anchor had been tattooed on the forearm of a young man five years earlier; about one year later, the warts appeared. Examination demonstrated about 20 warts; 19 of the 20 warts were scattered on the indigo lines of the tattoo [2].

**Table 1:** Tattoo-associated verruca vulgaris: epidemiology features A, age (years); C, case; Br, Brazil; Cr, Croatia; En, England; G, gender; Ge, Germany; HB, hepatitis B virus; HC, hepatitis C virus; HIV, human immunodeficiency virus; HPV, human papillomavirus; HT, human papillomavirus testing; IC, immunocompetent; In, India; It, Italy; M, man; MA, Mexican American; Na, nationality; NS, not stated; PT, professional tattoo; RT, refused testing; Sp, Spain; Sy, syphilis; TD, tattoo duration (number of months after tattoo placement that verruca vulgaris lesion appeared); US, United States; W, woman, Y, young; +, positive or present; -, absent or negative or no. ^_a_^Case 19: an investigator (surgeon captain RWB Scutt from Gosport, England) had surveyed 923 patients to determine the incidence of jaundice in tattooed individuals; at least one patient had warts on their tattoo [2]. Cases 20-27: over a nine-year period (2007-2016), a French investigator observed eight viral warts and one seborrheic keratosis on tattoos; the data were presented as a summary of the findings. The nine patients (six men and three women) ranged from 20 to 47 years (mean, 28 years) and were French (eight patients) or Finn (one patient). The warts appeared 6 to 18 months after the patient acquired the tattoo; the tattoos had been present from one to five years [10]. ^b^The patient “was tattooed in Port Elizabeth by an Italian with Indian ink.” ^c^The warts “had appeared in a tattoo soon after its application.” ^d^A positive reaction for HPV could not be demonstrated with in situ hybridization using a pan HPV probe and polymerase chain reaction. ^e^HPV type 27 was detected (by polymerase chain reaction using a nested primer system, cloning, and sequencing) and verified (by analysis of the obtained sequence information). ^f^The patient has HIV infection and was receiving antiretroviral therapy (zidovudine, lamivudine, ritonavir, and darunavir); his viral load is undetectable, and his CD4 cell count is 582/mm^3^.

C^a^	A	Na	G	IC	HB	HC	HIV	Sy	HT	PT	TD	Reference
1	Y	US	M	+	NS	NS	NS	NS	NS	+	12	[2] C1
2	Y	En	M	+	NS	NS	NS	NS	NS	-^b^	1	[2] C2
3	17	Sp	W	+	-	-	-	NS	NS	+	2	[3] C8
4	18	In	M	+	-	-	-	NS	NS	-	12	[4]
5	21	US	M	+	NS	NS	NS	NS	NS	+	1^c^	[2] C4
6	24	MA	M	+	NS	NS	NS	NS	NS	+	21	[3] C2
7	24	It	M	+	-	-	-	NS	NS	+	54	[5] C1
8	25	Sp	M	+	NS	NS	NS	NS	NS	+	NS	[6]
9	27	US	M	+	NS	NS	RT	NS	NS	-	94	[3] C5
10	29	Ge	M	+	-	-	-	NS	+^d^	+	96	[3] C7
11	31	Sp	M	+	NS	NS	NS	NS	NS	+	12	[7]
12	31	US	W	+	-	-	-	NS	+^e^	+	24	[3] C10
13	32	Cr	M	+	-	-	-	NS	NS	+	26	[3] C6
14	33	Br	M	-^f^	NS	NS	+	NS	NS	+	72	[8] C2
15	35	It	M	+	-	-	-	NS	NS	+	32	[5] C2
16	39	Br	M	+	NS	NS	NS	NS	NS	+	12	[8] C1
17	44	US	W	+	NS	NS	NS	NS	NS	+	252	Current report
18	66	US	W	+	NS	NS	NS	NS	NS	+	19	[9]

**Table 2:** Tattoo-associated verruca vulgaris: clinical features Bx, biopsy confirmation of verruca vulgaris diagnosis; C, case; LN2, liquid nitrogen cryotherapy; Ref, reference; YAG, yttrium aluminum garnet; +, positive or present; -, absent or negative or no. ^a^Case 19: the figure legend read “Warts in a tattoo.” The tattoo was black—yet the location could not be determined from the image—with at least 20 warts; three or more warts were present in adjacent normal skin [2]. Cases 20-27: The number of warts within the tattoo ranged from 1 (two patients) to 12 to more than 100 (six patients); two patients also had warts in the adjacent normal skin, and one of these patients confirmed that the wart was present prior to obtaining the tattoo. The tattoo was in the black ink (seven patients) or a multicolored area (one patient). The diagnosis was established clinically (seven patients) or by biopsy (one patient). Management included either abstention, cryotherapy, or topical therapy (retinoid or fluorouracil). Follow-up, in three patients, showed that the warts resolved after cryotherapy or topical retinoid [10]. ^b^The patient also had current or a history of human papillomavirus infection that was not associated with their tattoo: current perianal (case 8) or genital (case 14) condyloma acuminata or a prior wart on the back (case 18). ^c^Dermoscopy showed skin-colored homogenous papillomatous papules in one patient (case 11). In the other patients, in vivo dermoscopy showed either “a nonspecific appearance with isolated spots of desquamation” (case 14) or “some projections of similar diameter and length in a ‘knob’ pattern over the papules, with thrombosed glomerular capillaries, giving the appearance of red spots on the lesion’s surface” (case 16) and ex vivo dermoscopy showed either “a slightly thickened epidermis with discrete papillomatosis and the presence of pigment in the dermis” (case 14) or “a papillomatous verruciform appearance in the epidermis with underlying pigment in the dermis” (case 16).

C^a^	Number of lesions	Site	Tattoo color	Bx	Treatment	Ref
1	20	Forearm	Indigo	-	Not stated	[2] C1
2	38	Not stated	Indian ink	-	Not stated	[2] C2
3	13	Left leg	Dark blue	+	Curettage (12 lesions); none recur at two-month follow-up	[3] C8
4	Multiple	Left forearm	Black	+	Electrocautery planned; no results stated	[4]
5	7	Left arm	Black	+	None; patient concerned about tattoo cosmetics	[2] C4
6	Countless	Left scapula	Black	+	LN2; ablated most lesions; yet, associated postinflammatory hypopigmentation	[3] C2
7	Greater than 400	Right arm	Dark color, Red	-	Squaric acid dibutyl ester contact immunotherapy; warts completely regressed within eight weeks; none recur at 10-month follow-up	[5] C1
8	Multiple	Left dorsal hand^b^	Black, Green	-	Not stated	[6]
9	Numerous	Right scapula	Dark blue	+	LN2; clearing of lesions; yet residual hypopigmentation and mild distortion of the tattoo	[3] C5
10	Numerous	Left arm	Dark blue	+	5% imiquimod cream; warts unchanged	[3] C7
11	Multiple	Abdomen	Black	+^c^	Not stated	[7]
12	Multiple	Right dorsal foot	Green, Red	+	5% imiquimod cream; minimal improvement	[3] C10
13	Greater than 104	Left scapula	Black	+	Not stated	[3] C6
14	Multiple	Left arm^b^	Black	+^c^	Not stated	[8] C2
15	Greater than 80	Left arm	Dark dye	-	Squaric acid dibutyl ester contact immunotherapy; warts completely regressed within 10 weeks; none recur at 10-month follow-up	[5] C2
16	Numerous	Left calf	Black, Blue	+^c^	Not stated	[8] C1
17	1	Left lateral distal leg	Black	+	Biopsy removed most of the wart; no additional treatment	Current report
18	Confluent	Left and right eyebrows^b^	Black, Dark grey	+	Ablative erbium:YAG laser (one session), 80% resolution; then, 5% imiquimod cream (daily for 12 weeks), complete resolution; none recur at 20-month follow-up	[9]

It was not until 24 years later that a second patient (a young Englishman) with infective warts on his tattoo was reported, with no prior or current history of other warts, and he developed warts (one that was larger and bleeding and 37 smaller lesions) that began to appear, along the lines of a tattoo, one month after receiving the tattoo created with Indian ink by an Italian in Port Elizabeth six months earlier [2].

More than half a century passed after the second patient presented with warts on his tattoo, and a 21-year-old man (with no other verruca) presented with seven warts in the black pigment of a rose tattoo on his left arm; the tattoo was acquired two years earlier and the biopsy-confirmed warts appeared soon after its application. The patient and his father were both overwrought by the destruction of the tattoo at the biopsy site, and a mutually agreeable course of action could not be arrived at. Therefore, the viral lesions were not treated [2].

Since 1961, an additional 24 patients with verruca vulgaris, including the woman in this paper, have been reported. The papers for nine of these individuals with verruca vulgaris on their warts did not provide all the patient-specific epidemiologic or clinical details. However, complete epidemiologic features and clinical information were available for at least 18 of the patients whose tattoos contained verruca vulgaris (Tables 1, 2, cases 1-18) [2-10].

The age at diagnosis for 14 men and 4 women with verruca vulgaris on their tattoos ranged from 17 to 66 years (median, 28 years). The men ranged in age from 18 to 39 years (median, 25 years). The women ranged in age from 17 to 66 years (median, 38 years).

The development of a verruca vulgaris in a tattoo is a worldwide phenomenon. Most of the individuals were either from the United States (seven patients, including the Mexican American man) or France (at least seven, and possibly eight, patients). Spain (three patients) and Brazil, England, or India (two patients) were followed in frequency of reported nations. Single patients were noted from Croatia, Germany, Italy, and possibly Finland.

One of the patients, a 33-year-old man, was immunosuppressed; he was infected with human immunodeficiency virus and receiving antiretroviral therapy [8]. Seven other patients had additional evaluation for systemic viral infections such as hepatitis B, hepatitis C, and human immunodeficiency virus; all results were negative. None of the patients with tattoo-associated verruca vulgaris were tested for syphilis.

Additional human papillomavirus testing (including in situ hybridization and/or polymerase chain reaction) was performed for two patients. Human papillomavirus type 27 was detected and verified in a 31-year-old woman (case 12) [3]. However, testing failed to show a positive reaction for human papillomavirus in a 29-year-old man (case 10) [3].

The tattoo was done by a professional tattoo artist for 15 of the patients. One young man from England had his tattoo applied “by an Italian” in Port Elizabeth (which is now known as Gqeberha), a city on the Algoa Bay of the Indian Ocean in South Africa’s Eastern Cape Province (case 2) [2]. The tattoo was performed by an amateur for two individuals; the first was an 18-year-old Indian man who had a tattoo of the face of the revered revolutionist Ernesto “Che” Guevara etched on the ventral aspect of his forearm “with black dye by a nonprofessional person” (case 4) [4]. The second was a 27-year-old, currently incarcerated, prisoner whose “… tattoo was performed by an amateur using a staple to implant the pigmented material consisting of ink from a ballpoint pen, melted plastic, and the artist’s saliva which was used to thin the material to the right consistency” (case 9) [3].

The duration of time between the patient receiving the tattoo and the development of warts on the tattoo varied from one month to 21 years (median, 21 months). In men, the latency period ranged from one month to eight years (median, 21 months). In women, the warts appeared two months to 21 years (median, 22 months) after receiving their tattoo.

A closer examination of the latency period between tattoo acquisition and wart development shows that 41% of individuals (seven of 17 patients) presented with warts within one year of receiving their tattoo. Indeed, 70% of individuals (12 of 17 patients) noticed the warts within three years after their tattoos were received. The remainder of the patients experienced the onset of warts either between five to eight years after acquiring their tattoo (24%, four of 17 patients) or after 21 years (6%, one of 17 patients).

Similar trends are observed when the duration of time between tattoo placement and the development of warts on the tattoo are examined for each gender. Tattoo-associated warts developed in 46% of men (six of 13 patients) within one year after tattoo placement; by three years, 69% of men (nine of 13 patients) had warts and the viral lesions appeared in the remaining 31% of men (four of 13 patients) between five to eight years after they received their tattoo. Tattoo-associated warts developed in 25% of women (one of four patients) within one year after tattoo placement; however, by two years, 75% of women (three of four patients) had warts and the viral lesions appeared in the remaining 25% of women (one of four patients) 21 years after they received their tattoo. 

The number of verruca vulgaris ranged from one to more than 400 (median, 17). Descriptive terms were also used to describe the number of warts. These included multiple, numerous, confluent, and countless.

The tattoos were mostly located on the extremities (70%, 12 of 17 patients). The upper extremity was the site of eight tattoos (47%) that were located on either the arm (five tattoos), the forearm (two tattoos), or the hand (one tattoo). The lower extremity was the site of four tattoos (23%) that were located on either the leg (two tattoos), the calf (one tattoo), or the foot (one tattoo). The remaining tattoos were located on the scapula (18%, three tattoos), the abdomen (6%, one tattoo), and the eyebrows (6%, one tattoo).

Most of the patients had verruca vulgaris restricted only to the tattoo without any involvement of the adjacent normal skin. However, at least six patients (cases 1, 10, 14, 19, and 20-27) had one or more warts on skin that was not tattooed; indeed, one of these individuals confirmed that the wart had been present prior to obtaining the tattoo (cases 20-27). In addition, three of the patients had human papillomavirus-associated lesions that were not only present either prior to (on the back, case 18) or concurrent with (on the perianal, case 8, or the genital, case 14, area) the verruca in their tattoo but also appearing at a distant location from the tattoo-associated warts.

All 27 patients had verruca vulgaris in the dark-colored portion of their tattoo. The most common pigment color was black (19 patients). Some of the warts occurred in the blue (four patients), green (two patients), dark (not otherwise specified, two patients), and red (two patients) tattoo ink. Individual patients had warts in the tattoos that were grey, indigo (dark purplish blue), or multicolored. The verruca vulgaris were only found in one color of the tattoo in 22 patients; however, five of the patients had warts that appeared in more than one color of their tattoo: black and blue, black and dark grey, black and green, dark (not otherwise specified) and red, and green and red.

Several hypotheses have been suggested regarding the development of human papillomavirus on a tattoo. Viral contamination of the needles or contaminated tattoo dye or both are the favored mechanisms of introduction of the pathogen into the skin; a wart on the ungloved hand of the tattoo artist has also been suggested as a potential source of the virus [3,9]. However, an unnoticed verruca at the site of tattoo placement with subsequent traumatic spread of the virus during tattoo inoculation has also been postulated; indeed, one patient with a wart in the vicinity, but not actually in the tattooed area, confirmed that the wart was present prior to acquiring the tattoo [10].

The saliva of the tattoo artist has also been suggested as the source of human papillomavirus in the recipient’s tattoo [9]. The potential possibility of this mode of transmission was more likely when the saliva of the tattooist was the sole lubricant used during the inoculation of the tattoo [2]. However, this practice was still instituted in the mid-1980s, when a 19-year-old man had his tattoo performed by an amateur; saliva from the tattoo artist was used to thin the pigmented material (which consisted of a mixture of melted plastic and ballpoint pen ink) to the appropriate consistency so that it could be implanted with a staple. Nearly eight years later, verruca vulgaris appeared in the dark blue lines outlining the wings and body of a butterfly and lettering of a name on his right scapula (case 9) [3].

Another postulated mechanism for the development of human papillomavirus infection (including both verrucae vulgaris and verruca plana) in tattoos may be related to the development of a cutaneous immunocompromised district, a localized skin area with altered immunity. The trauma associated during the inoculation of the tattoo or the black or dark pigmented dye or both may have created the immunocompromised district localized to those specific cutaneous sites occupied by the ink in the tattoo. The alteration in either humoral immunity or cell-mediated immunity or both provides an increased propensity for previously existing or concurrently inoculated (during the application of the tattoo) or subsequently acquired human papillomavirus infection to eventually manifest as clinical warts [3,10-12].

The onset of warts within the cutaneous immunocompromised district may occur spontaneously or following an exogenous stimulus such as ultraviolet radiation. Indeed, a 32-year-old man developed multiple warts, confined to his tattoo, two weeks after experiencing an acute sunburn to his back (on which the tattoo was located) and shoulders. The previously lesion-free tattoo had been present for 2.5 years and had been done by a professional tattoo artist (case 13) [3].

A diagnosis of wart was based only on the morphologic appearance of the new lesion that appeared on the tattoo for 44% of the individuals (12 of 27 patients). Dermoscopy of the intact lesion (three patients) and biopsy of the lesion (two patients as a research investigation) were performed (Table 2, cases 11, 14, and 16) [7,8]. Including the three patients who had dermoscopic evaluation of their intact lesions, the suspected clinical diagnosis of verruca vulgaris was confirmed by microscopic evaluation of the tissue from a biopsy of the lesion in 54% of the individuals (15 of 27 patients).

The treatment of the verruca vulgaris was described for 11 of the patients (Table 2); it was either declined (one patient), immunologic (four patients), or procedural (six patients). A 21-year-old man’s tattoo of a rose on his left arm contained seven warts; following the biopsy of one wart which measured 4 mm in diameter, he and his father “both were overwrought by the destruction of the tattoo at the biopsy site.” Thereafter, “a mutually agreeable course of action could not be arrived at” and the patient did not proceed with treatment of the residual warts (case 5) [2].

Immunologic management included either topical application of 5% imiquimod cream or squaric acid dibutyl ester. Two men’s warts were unsuccessfully treated with imiquimod cream monotherapy. A 29-year-old man (case 10) with numerous warts on his left arm tattoo observed no change, and a 31-year-old man (case 12) with human papillomavirus type 27 in his multiple warts on the right dorsal foot only noted minimal improvement after treatment with topical imiquimod [3].

Two men, a 24-year-old with at least 400 warts on his right arm tattoos (case 7) and a 35-year-old man with at least 80 warts on left arm matryoshka (little matron) doll tattoo (case 15), had complete resolution of their viral lesions within 8 or 10 weeks, respectively, after initiating treatment with squaric acid dibutyl ester contact immunotherapy; there were no scars, no hypopigmentation, no hyperpigmentation, and no recurrence over a 10-month follow-up period. Sensitization was performed by applying 100 ml of 2% squaric acid dibutyl ester to an uninvolved site (10-cm^2^ area on the volar aspect of the uninvolved arm) for 48 hours. Weekly treatment, applying 100 ml of 0.01% squaric acid dibutyl ester directly on the warts, was initiated two weeks after sensitization [5].

Procedural management of the warts included the biopsy, cryotherapy, curettage, electrocautery, and laser therapy combined with topical imiquimod. The patient in this report, a 44-year-old woman who had a prolonged latency period of 21 years between acquiring her tattoo and the appearance of the wart on the tattoo’s green ink, did not want to proceed with any additional treatment (case 17). Most of the wart had been removed during the biopsy procedure using a punch tool; she did not return for scheduled follow-up.

Cryotherapy with liquid nitrogen was successfully used to treat two patients: a 24-year-old man with a large tattoo of a voluptuous mermaid on his left shoulder that had countless warts (case 6) and a 27-year-old man with a butterfly and name tattooed on his right scapular area that had numerous warts (case 9). There was ablation of most or all warts, respectively. However, both men had posttreatment hypopigmentation, and the older man also had mild distortion of his tattoo [3].

Curettage was successfully used to treat tattoo-associated warts. A 17-year-old woman had 13 warts that appeared on the dark blue areas of the tattoo on her left leg three months after it was received (case 3). After a biopsy of one of the lesions established the diagnosis, the remaining 12 warts were curetted; at two-month follow-up, there was no recurrence [3].

Electrocautery was scheduled for the treatment of multiple warts on a tattoo. The lesions had appeared on the tattoo of Che Guevara’s face on the left forearm of an 18-year-old man (case 4). However, at publication of the report, follow-up after the planned procedure was not provided [4].

Combination therapy, ablative erbium:yttrium aluminum garnet (YAG) laser (2,940 nm) and 5% imiquimod cream, successfully treated the cosmetic tattoo-associated confluent verruca vulgaris on both eyebrows of a 66-year-old woman (case 18). Her eyebrow tattoos had initially been performed by a professional artist 10 years prior and again (for redarkening) two years earlier; within five months of the second tattoo session, the warts appeared. After one laser session, 80% of the verruca was ablated; complete resolution of the residual verruca occurred after once daily application of imiquimod cream for 12 weeks. Follow-up at 20 months demonstrated that clearance of the verruca was maintained [9].

There may be a role for stressing preventative intervention with regards to decreasing the incidence of warts on tattoos. Indeed, an increasing number of reports of skin infections, not only viral infections such as warts but also bacterial, fungal, and mycobacterial infections, have been observed in individuals undergoing the tattooing process during recent years. Measures to ensure prevention of human papillomavirus infections during the acquisition of tattoos have been incorporated; the health surveillance criteria for tattoo parlors have been revised by the United States Food and Drug Administration in conjunction with the Centers of Disease Control and Prevention [8]. In addition, enhanced efforts to maintain sterility of the instruments and dyes used for tattooing as well as the skin surface intended for tattoo placement are essential [4]. 

Tattoo-associated verruca plana have been described in 14 patients ranging in onset age from 20 to 48 years (median, 30 years) (Table 3) [1,3,11-18]. The flat warts were diagnosed at ages ranging from 20 to 48 years (median, 33 years) in the five men and between the age of 21 and 39 years (median, 27 years) in the three women. The patients were from several different countries: the United States (two patients) and one patient from Canada, China, Germany, Korea, Netherlands, or Turkey.

**Table 3:** Tattoo-associated verruca plana: epidemiology features A, age (years); C, case; Ca, Canada; Ch, China; DNA, deoxyribonucleic acid; G, gender; Ge, German; HB, hepatitis B virus; HC, hepatitis C virus; HIV, human immunodeficiency virus; HPV, human papillomavirus; HT, human papillomavirus testing; IC, immunocompetent; Ko, Korea; M, man; Na, nationality; NS, not stated; PT, professional tattoo; Sy, syphilis; TD, tattoo duration (number of years after tattoo placement that verruca plana lesion appeared); Tu, Turkey; US, United States; W, woman, +, positive or present; -, absent or negative or no. ^a^Case 8: investigators reported human papillomavirus-induced cutaneous verruca plana (flat warts) in a healthy 21-year-old woman from the Netherlands [18]. Case 9-14: over a 10-year period (2000-2010), seven cases (including case 4) of verruca located in black tattoo ink were observed in one solo private practice in the United States [11,12]. ^b^Human papillomavirus type 6B DNA was detected in the lysate of the wart specimen when paraffin-embedded sections of formalin-fixed tissue were studied by using an HPV polymerase chain reaction technique. ^c^Immunohistochemical analysis using monoclonal murine anti-HPV antibody detected positive nuclear staining for HPV in nearly all the keratinocytes in the stratum corneum and a few of the cells in the stratum granulosum of the wart biopsy specimen. Subsequently, the presence of cutaneous beta 1-human papillomavirus type 47 DNA was demonstrated by virus polymerase chain reaction and consecutive DNA sequencing.

C^a^	A	Na	G	IC	HB	HC	HIV	Sy	HT	PT	TD	Reference
1	20	Tu	M	+	-	-	-	NS	NS	-	0.5	[13]
2	21	US	M	+	-	-	-	-	NS	+	0.5	[14]
3	27	Ch	W	+	NS	NS	NS	NS	NS	+	2	[15]
4	33	US	M	+	NS	NS	NS	NS	+^b^	+	10	[3] C4
5	36	Ca	M	+	-	-	-	NS	NS	+	>19.5	[16]
6	39	Ko	W	+	-	-	-	-	NS	-	1	[17]
7	48	Ge	M	+	NS	NS	NS	NS	+^c^	+	12	[1]

All the patients who developed flat warts on their tattoo were immunocompetent. Additional testing for other viral and spirochete infections was also performed. None of the individuals tested had infection from hepatitis B (four patients), hepatitis C (four patients), human immunodeficiency virus (four patients), and syphilis (two patients).

Five of the tattoos were performed by a professional tattooist. However, two of the patients’ tattoos were obtained from amateurs. 

The duration of time between obtaining the tattoo and the development of the flat warts varied from six months to more than 19.5 years (median, two years); however, more than half of the individuals (four of seven patients) noticed that the wart appeared within two years after receiving their tattoo in contrast to 43% of the individuals (three of seven patients) with prolonged latency periods of 10 or more years. The warts appeared between 0.5 and greater than 19.5 years (median, 10 years) in the men and between one and two years (median, 1.5 years) in the women.

The number of flat warts was most frequently described as multiple (four patients). A 21-year-old man had 150 lesions, a 33-year-old man had 120 lesions, a 36-year-old man had 100s of verruca plana, and a 48-year-old man had more than two lesions (Table 4) [1,3,11-18]. 

**Table 4:** Tattoo-associated verruca plana: clinical features Bx, biopsy confirmation of verruca plana diagnosis; C, case; LN2, liquid nitrogen cryotherapy; Ref, reference; +, positive or present. ^a^Case 8: investigators reported human papillomavirus-induced cutaneous verruca plana (flat warts) presenting as multiple, 1-4 mm, skin-colored papules following the ink pattern of a feather-shaped tattoo [18]. Case 9-14: 181 warts (from five—including case 4—of these patients) were localized in the black ink seven times more than in other colored ink and normal skin [11,12]. ^b^The verruca plana initially appeared above the tattooed area on the left anterolateral leg and dorsal foot; however, it eventually involved the tattoo which was performed to conceal a scalded scar. ^c^The verruca plana initially appeared as a solitary lesion on the right eyebrow; subsequently, over the next year, the verruca plana spread to her entire face. ^d^The number of lesions and/or the color of the tattoo was determined by viewing the clinical image included in the report.

C^a^	Number of lesions	Site	Tattoo color	Bx	Treatment	Ref
1	Multiple	Right arm	Dark blue	+	Retinoic acid cream (1% topical); no improvement	[13]
2	150	Left chest	Black	+	Not stated	[14]
3	Multiple	Left leg^b^, Left foot	Black	+	LN2 for three cycles and imiquimod cream (5% topical) for five months; not recur at five-month follow-up	[15]
4	120	Right upper arm	Black	+	Curettage—with excellent healing—at week 0, 2, 10, and 62; the number of warts treated at each time was 119, a few, 5, and 10	[3] C4
5	100s	Right arm, Right forearm	Black	+	LN2 for one cycle; no improvement	[16]
6	Multiple	Right eyebrow^c^	Black^d^	+	Imiquimod cream (5% topical); no improvement	[17]
7	Greater than two^d^	Left forearm, Right forearm	Black^d^	+	10% sinecatechins ointment; response to therapy not stated	[1]

The tattoo was mostly located on an extremity, either the arm (two men) or the arm and forearm (one man) or both forearms (one man) or the leg and foot (one woman). The warts were located on the chest of one man. The site of the final patient was initially her right eyebrow prior to the viral lesions spreading and affecting her entire face.

The tattoo pigment containing the verruca plana was dark in all the patients. It was black inked tattoo in six individuals. The last patient, a 20-year-old man, had his flat warts on a dark blue tattoo. Indeed, some of the investigators postulated that an alteration in the local immunity of the tattoo, a cutaneous immunocompromised district, was created by the black tattoo ink [3,11,12].

The diagnosis of verruca plana was confirmed by evaluation of a skin lesion biopsy in all the patients. Additional human papillomavirus testing was performed in two patients. Polymerase chain reaction detected human papillomavirus type 6B deoxyribonucleic acid (DNA) in the lysate from the paraffin-embedded sections of the formalin-fixed tissue of the wart biopsy specimen from the right upper arm of the 33-year-old man [3,11,12]. Virus polymerase chain reaction and consecutive DNA sequencing also established the presence of human papillomavirus type 47 DNA in the wart on the forearm of a 48-year-old man [1].

One of the patients, the 27-year-old Chinese woman, had the warts on her leg successfully treated with combination therapy. Three monthly treatment sessions using liquid nitrogen cryotherapy were combined with topical 5% imiquimod cream for five months. There was not only marked improvement in her lesions but also no recurrence during the subsequent follow-up period of five months [15].

Another patient, a 33-year-old man, experienced excellent healing after serial curettage sessions. At week zero, 119 warts were curetted; two weeks later, a few more lesions were treated. Subsequently, 5 and 10 verruca plana were curetted at follow-up visits two and a half months and one year later, respectively [3,11,12].

Topical therapy was initiated for the planar warts on the forearms of a 48-year-old man. The investigators had him apply 10% sinecatechins ointment; this is a green tea leaf extract approved for the management of genital warts. However, the researchers did not state the outcome of this treatment [1].

None of the other patients had successful management of their verruca plana. One treatment session of cryotherapy using liquid nitrogen resulted in no improvement. Similarly, topical monotherapy with either 1% retinoic acid cream or 5% imiquimod cream did not demonstrate any improvement.

Epidermodysplasia verruciformis is a rare autosomal recessive condition with not only an increased susceptibility to human papillomavirus infections, most commonly human papillomavirus types 5 and 8, but also an associated high risk of developing skin cancer. Acquired epidermodysplasia verruciformis is a condition that occurs in individuals with altered cell-mediated immunity; in addition to patients with human immunodeficiency virus infection, it has also been observed in patients with lymphoma, systemic lupus erythematosus, immunoglobulin M (IgM) deficiency, and individuals who are receiving immunosuppressive therapy following solid organ transplants [19,20].

Human immunodeficiency virus-associated acquired epidermodysplasia verruciformis has been observed localized to the tattoos of two men: a 29-year-old from the United States and a 44-year-old from Spain (Table 5) [19,20]. Both men presented with multiple viral lesions restricted to either the dark grey or the black and red inked portions of their tattoos (Table 6) [19,20]. The tattoos were located either on the upper back of the 29-year-old man or the left arm and forearm of the 44-year-old man. 

**Table 5:** Tattoo-associated acquired epidermodysplasia verruciformis: epidemiology features A, age (years); C, case; G, gender; HT, human papillomavirus testing; M, man; Na, nationality; NS, not stated; PT, professional tattoo; Ref, reference; Sp, Spain; TD, tattoo duration (number of years after tattoo placement that acquired epidermodysplasia verruciformis lesions appeared); US, United States; +, positive or present. ^a^Human immunodeficiency virus was diagnosed eight months prior to presentation of the acquired epidermodysplasia verruciformis. He has a medical history of cystic fibrosis, eczema, and genital molluscum contagiosum. ^b^He presented with four months duration of redness, dryness, and pruritus at the tattoo periphery; the duration of time between acquiring the tattoo and the onset of the erythematous, eczematous linear plaques of acquired epidermodysplasia verruciformis was not stated.

C	A	Na	G	Human immunodeficiency virus	HT	PT	TD	Ref
1	29	US	M	+^a^; CD4 count: 42 cells/mm^3^ (reference range, 500-1,200 cells/mm^3^); Viral load: 2,388 copies/ml (reference range, 20-10,000,000 copies/ml)	NS	NS	NS^b^	[19]
2	44	Sp	M	+; + antiretroviral treatment; Viral load: irregular control	NS	NS	NS	[20]

**Table 6:** Tattoo-associated acquired epidermodysplasia verruciformis: clinical features Bx, biopsy confirmation of acquired epidermodysplasia verruciformis diagnosis; C, case; Dm, dermoscopic; LN2, liquid nitrogen cryotherapy; NS, not stated; PT, professional tattoo; Ref, reference; +, positive or present. ^a^The tretinoin cream and the betamethasone dipropionate ointment were prescribed to be alternately applied topically on the tattoo lesions until resolution. ^b^Three sessions of photodynamic therapy, at three-week intervals, were performed. Following incubation for three hours with topical methyl aminolevulinate (MAL), the acquired epidermodysplasia verruciformis lesions were illuminated using a light-emitting diode (LED)-based red light source of 635 nm with a fluence of 37 J/cm^2^ per session. There was complete remission of all lesions and no recurrence at two-year follow-up.

C	Number of lesions	Site	Tattoo color	Dm	Bx	Treatment	Ref
1	Multiple	Upper back	Dark grey	NS	+	Tretinoin cream (0.1% topical) and betamethasone dipropionate ointment (0.05% topical)^a^	[19]
2	Multiple	Left arm, Left forearm	Black, Red	+	+	LN2: pain and no improvement; Photodynamic therapy^b^ for three sessions: complete resolution; not recur at two-year follow-up	[20]

The reports did not share whether the tattoos were created by an amateur or a professional. In addition, the latency period between the inoculation of the tattoo and the onset of epidermodysplasia verruciformis lesions was not described. Also, additional studies to identify the human papillomavirus type were not conducted.

Both men had a positive viral load for human immunodeficiency virus; one also had a low cluster of differentiation 4 (CD4) count, whereas the other was receiving antiretroviral treatment. The diagnosis of acquired epidermodysplasia verruciformis was established after an evaluation of the tissue biopsy from one of the lesions; dermoscopy was also performed on the viral lesions of the younger patient.

There are several different treatment modalities that have been used in the management of epidermodysplasia verruciformis. The younger man with epidermodysplasia verruciformis lesions on his upper back was instructed to alternately apply 0.1% tretinoin cream and 0.05% betamethasone dipropionate ointment until the viral lesions resolved. Posttreatment follow-up was not provided [19].

The older man was initially treated with cryotherapy using liquid nitrogen; this was not only painful but also did not demonstrate any improvement. However, he achieved a complete and sustained resolution of all his epidermodysplasia verruciformis lesions after three photodynamic therapy sessions each provided at three-week intervals. At his two-year follow-up examination, there had been no recurrence of the epidermodysplasia verruciformis lesions [20].

## Conclusions

Tattoos may be associated with adverse cutaneous events such as infection. A woman with a tattoo of 26 years duration presented with a verruca vulgaris that appeared five years earlier within the black ink of the tattoo. Human papillomavirus infection on a tattoo includes not only verruca vulgaris but also verruca plana and human immunodeficiency virus-associated acquired epidermodysplasia verruciformis. Hypotheses for the development of a wart on a tattoo are the use of contaminated instruments or ink during tattoo inoculation, transmission of the virus from the tattoo artist’s ungloved hand or saliva, a preexisting (albeit unrecognized) human papillomavirus lesion adjacent to or at the site of the tattoo, or postinoculation acquisition of the verruca at the site of the tattoo. The predilection for the viral lesion to occur in dark, and most commonly black, ink suggests the possibility that the ink predisposes to the development of a cutaneous immunocompromised district that enables the subsequent human papillomavirus infection to clinically manifest. Topical monotherapy with either a retinoid or an imiquimod was not efficacious in the management of the warts. Although cryotherapy with liquid nitrogen was not beneficial in some of the patients, the treatment effectively ablated most or all the lesions (when used as monotherapy or followed by topical application of 5% imiquimod cream) in other individuals; however, mild distortion of the tattoo and/or hypopigmentation occurred. Successful approaches to the management of tattoo-associated warts also included ablative erbium:YAG laser treatment followed by topical application of 5% imiquimod cream, curettage, photodynamic therapy, and squaric acid dibutyl ester contact immunotherapy.

## References

[1] Krecké N, Smola S, Vogt T, Müller CS (2017). HPV-47-induced and tattoo-associated verrucae planae: report of a case and review of the literature. Dermatol Ther (Heidelb).

[2] Long GE, Rickman LS (1994). Infectious complications of tattoos. Clin Infect Dis.

[3] Wanat KA, Tyring S, Rady P, Kovarik CL (2014). Human papillomavirus type 27 associated with multiple verruca within a tattoo: report of a case and review of the literature. Int J Dermatol.

[4] Chatterjee K, Roy A, Ghosh R, Barua JK, Halder S, Banerjee G (2017). The bumpy face of Che Guevara: an interesting case. Indian J Dermatol.

[5] Fania L, Sordi D, Pagnanelli G, CavanI A, Mazzanti C (2017). Tattoo and warts: efficacy of topical immunotherapy. Eur J Dermatol.

[6] Navarro-Vidal B, González-Olivares M, Aguado-Lobo M, Borbujo Martinez JM (2015). [Vulgaris verruca on a tattoo]. Med Clin (Barc).

[7] Linares-Gonzalez L, Navarro-Triviño F, Ruiz-Villaverde R (2020). Dragon warts. Sultan Qaboos Univ Med J.

[8] Veasey JV, Erthal AL, Lellis RF (2020). In vivo and ex vivo dermoscopy of lesions from implantation of human papillomavirus in tattoos: report of two cases. An Bras Dermatol.

[9] Nemer KM, Hurst EA (2019). Confluent verruca vulgaris arising within bilateral eyebrow tattoos: successful treatment with ablative laser and topical 5% imiquimod cream. Dermatol Surg.

[10] Kluger N (2017). Viral warts and seborrhoeic keratoses on tattoos: a review of nine cases. J Eur Acad Dermatol Venereol.

[11] Ramey K, Ibrahim J, Brodell RT (2016). Verruca localization predominately in black tattoo ink: a retrospective case series. J Eur Acad Dermatol Venereol.

[12] Ramey K, Ibrahim J, Brodell RT (2017). Creating immunocompromised districts. J Eur Acad Dermatol Venereol.

[13] Karadag AS, Bilgili SG, Onder S, Kosem M (2014). A case of verru plana on tattoo. J Pak Med Assoc.

[14] Baxter SY, Deck DH (1993). Tattoo-acquired verruca plana. Am Fam Physician.

[15] Chen YJ, Nabi O, Diao P, Wan RY, Li L (2020). Verruca plana on a tattoo: a case report. Medicine (Baltimore).

[16] Kirchhof MG, Wong SM (2019). Tattoos and human papilloma virus: a case report of tattoo-associated flat warts (verrucae planae). SAGE Open Med Case Rep.

[17] Jung JY, Shin HS, Won CH, Cho S (2009). Facial verruca plana that developed after semipermanent tattooing. Ann Dermatol.

[18] Hendrix N, Rijken F (2020). A woman with papules in a tattoo. (Article in Dutch). Ned Tijdschr Geneeskd.

[19] Jibbe A Snyder V, Fraga G, Aires D, Rajpara A (2020). Erythematous plaques on a tattoo. Cutis.

[20] Gracia-Darder I, Montis Palos MC, Ramos D, Saus C, Escalas Taberner J, Del Pozo Hernando LJ (2021). Acquired verruciform epidermodysplasia on a tattoo was successfully treated with photodynamic therapy. Photodiagnosis Photodyn Ther.

